# A service evaluation of ‘IDEAS’ – Modular treatment for youth with complex emotional needs

**DOI:** 10.1111/bjc.70034

**Published:** 2026-01-14

**Authors:** Annabel Harding, Franco Orsucci, Joanna Baines

**Affiliations:** ^1^ Department of Clinical Psychology and Psychological Therapies University of East Anglia Norwich UK; ^2^ Norfolk and Suffolk NHS Foundation Trust Norwich UK

**Keywords:** borderline personality disorder, complex emotional needs, emotional dysregulation, mental health, psychological intervention, psychological treatment

## Abstract

**Objectives:**

There is a crucial need for an evidence‐based intervention for young people presenting with moderate complex emotional needs (CEN). UK CEN care has been described as variable and poor quality, with its improvement a UK policy priority. Briefer‐versions of full‐programme therapy packages utilizing stepped‐care models offer a clear and contained pathway for this population, though their current availability in services is scarce. This service evaluation aims to evaluate the efficacy of ‘IDEAS’ as a piloted brief psychological intervention for young people aged 16–25 presenting with moderate CEN.

**Method:**

A pre‐test/post‐test design was used to explore clinical outcomes for participants and services via paired sample *t*‐tests, mixed‐model ANOVAS and frequency statistics.

**Results:**

Exploratory pre/post‐intervention analyses revealed significant reductions in mean scores for the severity of borderline personality disorder symptoms (*d* = .83), emotion regulation (*d* = 1.15) and overall wellbeing (reflecting improvement; *d* = .97) and increases in mean scores for quality of life (*d* = −.67), including level of satisfaction in one's quality of life and of therapeutic treatment received (*d* = −.65). These outcomes were maintained at 3‐month follow‐up, were little influenced by clinician training and supported readiness for discharge post‐intervention for more (68.65%) participants.

**Conclusions:**

The current evaluation provides preliminary evidence that IDEAS may offer a feasible, lower‐cost alternative to full‐programme treatment packages, with benefits for young people, services and Trusts. Given this evaluation of IDEAS being from a small, uncontrolled pilot, the findings are exploratory. Further evaluation is therefore warranted upon its larger‐scale implementation.

**Practitioner points**

- Complex emotional needs (CEN) describes the cluster of emotional and interpersonal difficulties frequently observed in people with a diagnosis of personality disorder.
- IDEAS is an eight‐session, one‐to‐one, brief psychological intervention for young people who present with moderate CEN.
- Early exploratory analyses revealed IDEAS to reduce symptom severity and increase quality of life, with outcomes maintained at three‐month follow‐up.
- IDEAS could offer a feasible and lower‐cost complement to full‐programme treatment packages, ensuring timely support to all levels of need and enhancing the CEN treatment pathway.

## INTRODUCTION

### Complex emotional needs

Complex emotional needs (CEN) is a recent, non‐diagnostic term describing the cluster of emotional and interpersonal difficulties frequently observed in people with a diagnosis of personality disorder (PD; Foye et al., 2022; Ledden et al., 2022). Coined by the National Institute for Health and Care Research (NIHR) Mental Health Policy Research Unit (MHPRU; NIHR MHPRU, 2024), this UK‐based term emerged in response to long‐standing controversy and stigma surrounding the ‘Personality Disorder’ label (Porter et al., 2025), including its history as a diagnosis of exclusion (National Institute for Mental Health England [NIMHE], 2003). Whilst its pseudo‐diagnostic nature has received criticism from both researchers and service‐users alike (Porter et al., 2025), its use represents a paradigmatic shift in mental health assessment that prioritizes understanding individual needs and life experiences over categorical symptom identification and depicting one's personality as a disorder. Both terms, CEN and PD, are used in this paper to note the specific patient population, as in alignment with the literature and collaborated with experts by experience.

Globally, an estimated 8% of the population experience difficulties consistent with CEN that meet PD diagnostic criteria (Botham et al., 2021), where prevalence in Great Britain is estimated at approximately 4.4% (Coid et al., 2006). PD itself is characterized by enduring, pervasive patterns of emotional and cognitive disturbance that shape self‐perception and interpersonal functioning (Winsper et al., 2015). A prominent subtype is borderline personality disorder (BPD), which is characterized by emotional dysregulation, impulsivity, suicidality and interpersonal dysfunction (Chapman, 2010). BPD affects .7%–2.7% of the general population and is associated with annual direct and indirect health‐care costs that are 16 times higher than matched controls (Hastrup et al., 2019). It is marked by instability across relationships, behaviour and mood (Chapman, 2019) and its diagnosis carries significant implications including high rates of psychiatric comorbidity (Shah & Zanarini, 2018), heavy service use (Meuldijk et al., 2017) and substantial treatment costs (Brettschneider et al., 2014).

Emotional dysregulation is a component of BPD (Chapman, 2019), which refers to an individual's reduced ability to regulate their emotional experiences through cognitive, behavioural, interpersonal and intrapersonal strategies (Gross, 2002; Gross & Thompson, 2007; Mansueto et al., 2022). It is argued to underlie many BPD symptoms, whereby impulsive behaviours (e.g., self‐harm and substance misuse) serve as maladaptive attempts to alleviate negative affect experienced (Brown et al., 2002; Kullgren, 1988; Linehan, 1993; Montgomery et al., 1989; Vollrath et al., 1996; Yen et al., 2002). This may be particularly prominent during adolescence and early adulthood, where such developmental stages are characterized by significant environmental pressure (Casey et al., 2010), ongoing neurobiological development, emotional reactivity and impulsive behaviour (Ahmed et al., 2015; Engel & Gunnar, 2020). This was particularly notable during the COVID‐19 pandemic, which exacerbated these developmental vulnerabilities by imposing emotional strain leading to overwhelm of negative emotions (Gerván et al., 2022). Prolonged school closures, social isolation, heightened household stress, economic strain and greater reliance on screens compounded such emotional strain on young people whilst simultaneously reducing their skills to manage (Khafif et al., 2021), resulting in ineffective and risky ways of coping.

Dialectical behaviour therapy (DBT) and mentalisation‐based therapy (MBT) are widely regarded as first‐line psychological treatments for individuals with CEN. However, the intensive staffing, specialist training and financial resources required for these full treatment packages often limit their feasibility for routine service delivery and are not always needed for all levels of clinical need. Shorter, adapted and modular formats have therefore emerged in response, such as DBT skills training; a briefer version of full‐programme DBT that is efficacious for transdiagnostic difficulties (e.g., emotional dysregulation) across diverse populations (Harvey et al., 2023; Wieczorek et al., 2021), within secondary‐care settings (Blackford & Love, 2011; Wright et al., 2020) and as a standalone treatment (Valentine et al., 2015). Such shorter, evidence‐based treatments have been noted to provide timely, more accessible and personalized care (Roberts et al., 2021). Further, by typically requiring fewer sessions, incorporating self‐help materials and being deliverable by clinicians without costly, specialized training (Bennett‐Levy et al., 2010), these interventions are reported to be more cost‐effective, better match service‐user preferences and thus be associated with lower dropout rates (Levy et al., 2018).

Such adaptations are vital given the current strain on NHS mental health services, which has led to long waits for assessment, lack of timely access to evidence‐based interventions and increasingly high thresholds for support. The presence of risk‐taking behaviour usually dictates the speed and level of support for individuals, with limited options for those presenting with less frequent and less risky behaviour. Therefore, young people presenting with moderate CEN face long waiting lists for assessment and further delays for appropriate support that is often generalist intervention solely focused on risk‐reduction and monitoring, with limited opportunity to develop collaborative psychological formulation to guide intervention.

There is therefore a crucial need to address this by developing an evidence‐based intervention for young people with moderate CEN; that is, CEN without a level of risk that would indicate the requirement for crisis support or longer‐term treatment. Although prevalence rates remain scarce, estimates suggest that between 1% and 3% of adolescents may meet criteria consistent with CEN (Chanen & Kaess, 2012; Winsper et al., 2015). Early intervention for this group has been noted as essential to prevent symptom escalation to crisis point (Royal College of Psychiatrists, 2024) and the reinforcement of maladaptive coping patterns into adulthood. However, UK CEN care has been reported as variable and poor quality (Dale et al., 2017) with concerns around quality and access (Foye et al., 2022), making it a UK policy priority for improvement within the NHS Long Term Plan (NHS England, 2019a) and NHS Mental Health Implementation Plan (NHS England, 2019b). Qualitative research notes that effective community CEN care must be person‐centred, flexible and co‐produced with experts by experience while providing training and supervision for clinicians (Foye et al., 2022). IDEAS is the response to this.

### The IDEAS (interventive dynamic emotion‐focused assessment and skills‐training) intervention

IDEAS is an eight‐session, one‐to‐one, brief psychological intervention developed within services to meet the needs of young people presenting with a primary needs type of CEN/personality disorder. Such individuals do not present with a level of risk that indicates the need for intensive, formal therapy or crisis support; though the stepped‐care model allows individuals to access longer‐term, formalized therapy (e.g., full‐programme DBT or MBT) if required post‐intervention.

Young people first work with their practitioner to develop an emotion‐focused formulation (Comprehend, Cope, Connect [CCC] formulation; Clarke, 2015) of their difficulties, before collaboratively selecting skills based on agreed deficits identified within the formulation. The intervention comprises primarily DBT‐based skills with complementary MBT techniques, such as emotion regulation, distress tolerance, interpersonal effectiveness, mindfulness, mentalisation and walking the middle path. All skills sessions follow a standard format of skill introduction, guided practice and homework review, supported by co‐produced workbooks and clinician guides.

Co‐production was at the core of IDEAS, as completed from the outset by a working group of experts‐by‐experience and People Participation Leads to ensure accessibility and acceptability, a strong service‐user voice throughout and individualisation of the intervention to prevent a ‘one‐size‐fits‐all’ treatment approach. All IDEAS clinicians must have received essential IDEAS training of 2‐day DBT skills training and internal training on the CCC formulation and IDEAS model; however, they can also have undergone full DBT training with DBT British Isles (intensive course or full postgraduate diploma). Finally, a clinical psychologist leads group clinical supervision of all clinicians to ensure careful clinical governance, fidelity monitoring to the IDEAS model and clinician skill development.

### Aims

The IDEAS pilot aimed to gather practice‐based evidence of the intervention prior to wider‐scale service implementation through the following aims:

1. To measure participant symptom change across time points (baseline, endpoint and 3‐month follow‐up).
2. To explore the influence of clinician training (fully DBT‐trained vs. non‐fully DBT‐trained) on participant symptom change from baseline to endpoint.
3. To explore post‐intervention service‐related outcomes (e.g., remaining open to services or discharge).
4. To explore the influence of clinician training on post‐intervention service‐related outcomes.

## METHOD

### Ethical considerations

Ethical approval was gained from the Trust's research and development team and the Faculty of Medicine and Health Sciences Research Ethics Subcommittee in England. Written consent to participate in the evaluation was provided by all participants via a consent form. Consent for evaluation was distinct from consent for the intervention, allowing participants to receive the intervention without participating in the evaluation. Confidentiality and anonymity were ensured by the dataset being pseudonymised via an identification number matched to each participant prior to it being shared for analysis.

### Design

This is a retrospective quantitative service evaluation using a quasi‐experimental one group pre‐test/post‐test design.

### Participants

Participants were service‐users aged 16–25 years old, held under two secondary‐care youth mental health teams in East Anglia, of whom received the IDEAS intervention. Both a diagnostic and needs‐led approach was used to select individuals for the intervention, which minimized restrictions associated with solely using diagnosis alone. Participants with a primary presenting need falling within the framework of the inclusion criteria (see Table 1) were selected via purposive sampling from the services' psychology waiting lists. Sample size was not predetermined; all eligible young people were offered IDEAS, with final numbers determined by clinician capacity.

**TABLE 1:** Participant inclusion and exclusion criteria.

Inclusion	Exclusion
Moderate CEN: Ongoing difficulties with managing emotions, relationships and sense of self that extend beyond everyday challenges and have a noticeable impact on daily life and functioning. These difficulties may include impulsive or risky behaviours (e.g., self‐injury or suicidal intent) used as coping strategies; however, the level of risk does not require crisis intervention or long‐term, specialist therapy such as full‐programme DBT or MBT. Diagnosis: Received a diagnosis of PD, meet diagnostic criteria for PD or present with comparable difficulties (e.g., emotional dysregulation, impulsivity, difficulty forming relationships, impaired self‐perception, risk of harm to self). Needs‐typing: Primary needs are best described within the ‘youth plus’ needs type (emerging difficulties beyond typical normal that requires early, lower‐level support) and/or are best described within the ‘emotional flux’ needs type (experience of recurrent emotional overwhelm managed through crisis behaviours). Coping behaviours: Maladaptive ways of coping at a mild‐to‐moderate risk level.	Co‐existing psychotic disorder/organic disorder. Coping behaviours: Maladaptive ways of coping at a moderate‐to‐high risk level (e.g., self‐injurious behaviours and/or active suicidal intent and attempts). A need for crisis support or formal, longer‐term therapy (e.g., full‐programme DBT or MBT).

*Note*: See Appendix A for full definitions of ‘youth plus’ and ‘emotional flux’ terms.

Of the 93 participants who were offered IDEAS and invited to participate in the evaluation, most were 20 years old (17%) and 24 years old (17%), female (89%) and of White ethnicity (78.5%). Ten individuals did not consent to the evaluation, of whom were predominantly female (80%), of White‐British ethnicity (90%) and aged 20 years old (30%). A further 22 participants did not complete IDEAS; again, predominantly female (82%), of White ethnicity (77%) and of similar age (18 years old; 27%).

**FIGURE 1:**
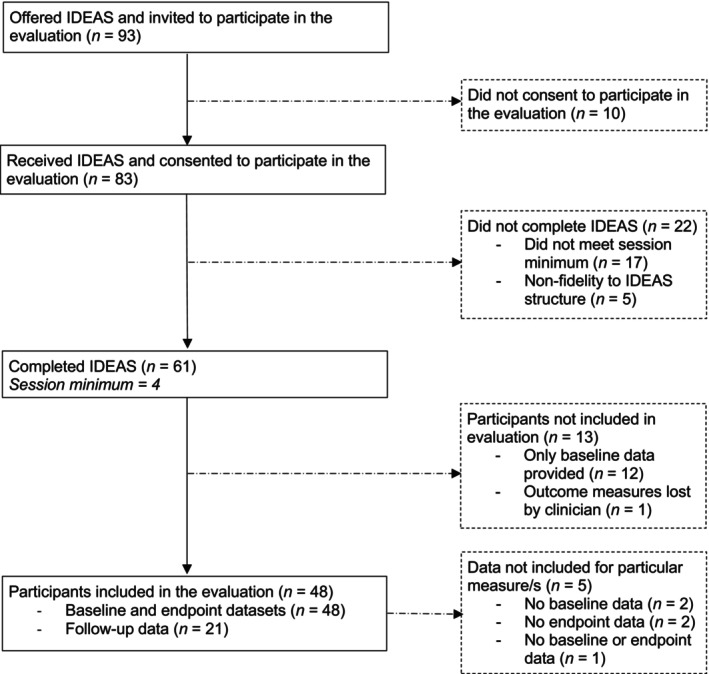
Participant flow throughout the intervention and evaluation. Session minimum' refers to the minimum number of sessions that each participant had to have had in order to complete IDEAS.

A total of 48 participants completed IDEAS, consented to participate in the evaluation and provided matched outcome measures; these participants comprised the evaluation's sample. Most participants were female (93.6%) and White‐British (77.1%). Few were of other ethnic backgrounds (10.41%), with 12.5% having no record of their ethnicity. A relatively even spread was seen across age groups, with the mean age being 20.8 years old (SD = 2.53). Of the total, 12 (25%) participants received IDEAS from a fully DBT‐trained clinician, where 36 (75%) participants received IDEAS from a non‐fully DBT‐trained clinician. See Figure 1 for full participant flow.

### Outcome measures

The following measures were administered at baseline (pre‐intervention), endpoint (post‐intervention) and follow‐up (3 months post‐intervention). All data were collected via self‐report.

#### The borderline symptom list‐shortened version (BSL‐23; Bohus et al., 2009)

A measure of borderline symptoms was used due to such symptoms reflecting core deficits in emotional regulation. The BSL‐23 is a briefer version of the BSL, which assesses the severity of symptoms of BPD that an individual presents with, as per the Diagnostic and Statistical Manual of Mental Disorders, fourth edition (DSM‐IV) criteria. Participants rate their experience of identified symptoms over the past week on a 5‐part Likert scale, where higher scores indicate a higher severity of BPD symptoms. This measure has a high internal consistency of .97 (Cronbach's alpha) and a (within 1 week) test–retest reliability of .82 (Bohus et al., 2009).

#### The clinical outcomes in routine evaluation – outcome measure (CORE‐OM; Evans et al., 2000)

The CORE‐OM assesses an individual's overall wellbeing, psychological difficulties, psychological functioning and risk. It comprises 34 items over four subscales that questions the individuals overall functioning over the past week, which is rated on a 5‐part Likert scale. Good reliability and an internal consistency of .75–.94 for the subscales are reported (Evans et al., 2000). A higher score reflects poorer overall psychological functioning.

#### The difficulties in emotion regulation scale – short form (DERS‐SF; Kaufman et al., 2016)

The DERS‐SF assesses difficulties across the four dimensions of emotion regulation: awareness and understanding of emotions, acceptance of emotions, ability to engage in goal‐directed behaviour and refrain from impulsive behaviour when experiencing negative emotions and access to emotional regulation strategies perceived as effective. It is widely used to explore this construct within adolescents and adults, with the short form providing a streamlined version that places less burden on the individual (Kaufman et al., 2016). The measure comprises 16 items, has a good internal consistency of .92 (Cronbach's alpha) and is strongly associated with the full 36‐item version (Gratz & Roemer, 2004). A higher score signifies a higher difficulty in emotion regulation.

#### The World Health Organization – quality‐of‐life (WHO‐QOL; The WHOQOL Group, 1998)

The WHO‐QOL assesses an individual's quality of life across four domains: physical health, psychological health, social relationships and environmental health and contains items related to quality of life and general health. The measure comprises 26 items and has a good internal consistency of .65–.93 (Cronbach's alpha; The WHOQOL Group, 1998). A higher score signifies a higher self‐reported quality of life.

#### Dialogue+

This measure is widely used in NHS health‐care and research to assess an individual's level of satisfaction with their quality of life and the treatment they have received. The former covers eight life domains and the latter covers three treatment domains, which are asked within 11 questions that are rated on 7‐point Likert scale. A higher score signifies an individual's higher satisfaction with their quality of life and treatment.

### Procedure

Participants underwent an initial assessment to confirm suitability for secondary care and for IDEAS. Session one completed the baseline outcome measures and CCC formulation (Clarke, 2015), followed by six skills sessions targeting the identified and agreed skills deficits. The final session reviewed the skills covered, completed the endpoint outcome measures and developed an onward post‐intervention plan. A 3‐month post‐intervention follow‐up was conducted by a clinician via telephone to repeat the outcome measures and review progress. Completion of IDEAS was defined as attending a minimum of four sessions to ensure meaningful engagement and symptom change. A total of 19 clinicians delivered IDEAS: 5 with essential IDEAS training and full DBT training and 14 with essential IDEAS training (including non‐full, 2‐day DBT training).

### Analysis plan

Data were analysed using Jamovi for macOS (version 2.5; The Jamovi Project, 2024). Some datasets were excluded from the analysis due to reasons outlined in Figure 1. All data removal was completed prior to the aggregation of the dataset and analysis. To explore participant symptom change (Aim 1), paired samples *t*‐tests were conducted on each outcome measure, which compared mean scores at baseline and endpoint and at endpoint and follow‐up. Adjusted Wilcoxon ranks scores were used for baseline and follow‐up for the CORE‐OM and DERS‐SF due to the Shapiro–Wilk test revealing violations of normality. To explore the effect of clinician training on participant symptom change (Aim 2), mixed‐model ANOVAS were conducted. Despite differing clinician group sizes, assumption checks revealed no violations, therefore confirming the ANOVAS to be robust against these non‐equivalent group sizes. Effect sizes were calculated for both *t*‐test and ANOVA analyses. To explore post‐intervention service‐related outcomes (Aim 3), descriptive statistics were run. Finally, to explore the impact of clinician training on post‐intervention service‐related outcomes (Aim 4), frequency statistics via a contingency table were completed, with a Fisher's Exact Test to allow for the unequal clinician group sizes. Service‐related outcomes were defined as the requirement for further psychological and/or medical intervention and/or risk management, or discharge. These data were collected from Trust Electronic Health Records and pseudonymised prior to analysis.

## RESULTS

### Participant symptom change

To explore Aim 1, paired samples *t*‐tests completed on each outcome from baseline to endpoint revealed statistically significant reductions in mean scores across the BSL‐23, CORE‐OM and DERS‐SF and increases in mean scores across the WHO‐QOL and DIALOGUE+, all with large effect sizes.

Of the 48 participants with complete baseline and endpoint data, 21 participants (43.75%) provided follow‐up data, resulting in a non‐response rate of 56.25%. Of these 21 participants, reductions in mean scores between baseline and follow‐up, and endpoint and follow‐up, were obtained across the BSL‐23, CORE‐OM and DERS‐SF. Further, increases were obtained at these timepoints across the WHO‐QOL and DIALOGUE+. Large effect sizes were revealed for the BSL‐23, CORE‐OM and DERS‐SF and small for the DIALOGUE+ and moderate for the WHO‐QOL from baseline to follow‐up. Small (or lesser) effect sizes were revealed across all measures between endpoint and follow‐up (see Table 2).

**TABLE 2:** Respective paired samples *t*‐test for baseline (pre‐intervention) and endpoint (post‐intervention) and baseline and follow‐up timepoints.

Measure	n	Mean scores (SD)			*t*‐statistic	*p*‐value	*d*
		Baseline	Endpoint	Follow‐up			
BSL‐23	48	59.70 (15.79)	40.00 (22.54)		5.75	**<.001**	.83
	21	61.60 (16.61)		36.90 (23.70)	4.33	**<.001**	.95 .37
CORE‐OM	47	81.60 (16.50)	56.30 (24.91)		6.62	**<.001**	.97
	21	83.0 (15.31)		55.70 (26.60)	218.0^a^	**<.001**	.89 .10
DERS‐SF	48	69.60 (8.72)	54.10 (14.40)		7.98	**<.001**	1.15
	21	69.30 (7.84)		54.00 (16.90)	2.15	**<.001**	.86 .05
DIALOGUE+	45	43.60 (9.45)	50.30 (9.58)		−4.37	**<.001**	−.65
	19	45.90 (9.04)		48.80 (15.80)	−.82	.42	−.19 .31
WHO‐QOL	47	72.90 (11.05)	82.60 (14.59)		−4.60	**<.001**	−.67
	20	72.80 (10.88)		81.50 (21.10)	−2.14	.05	−.47 .14

*Note*: The values on the first line relate to the baseline to endpoint *t*‐test, where the values on the second line relate to the baseline to follow‐up *t*‐test. *p*‐values in bold are statistically significant.

Abbreviation: *d*, effect size.

^a^ Wilcoxon‐signed rank test (*W*) and adjusted effect size.

### Participant symptom change by clinician training

**FIGURE 2:**
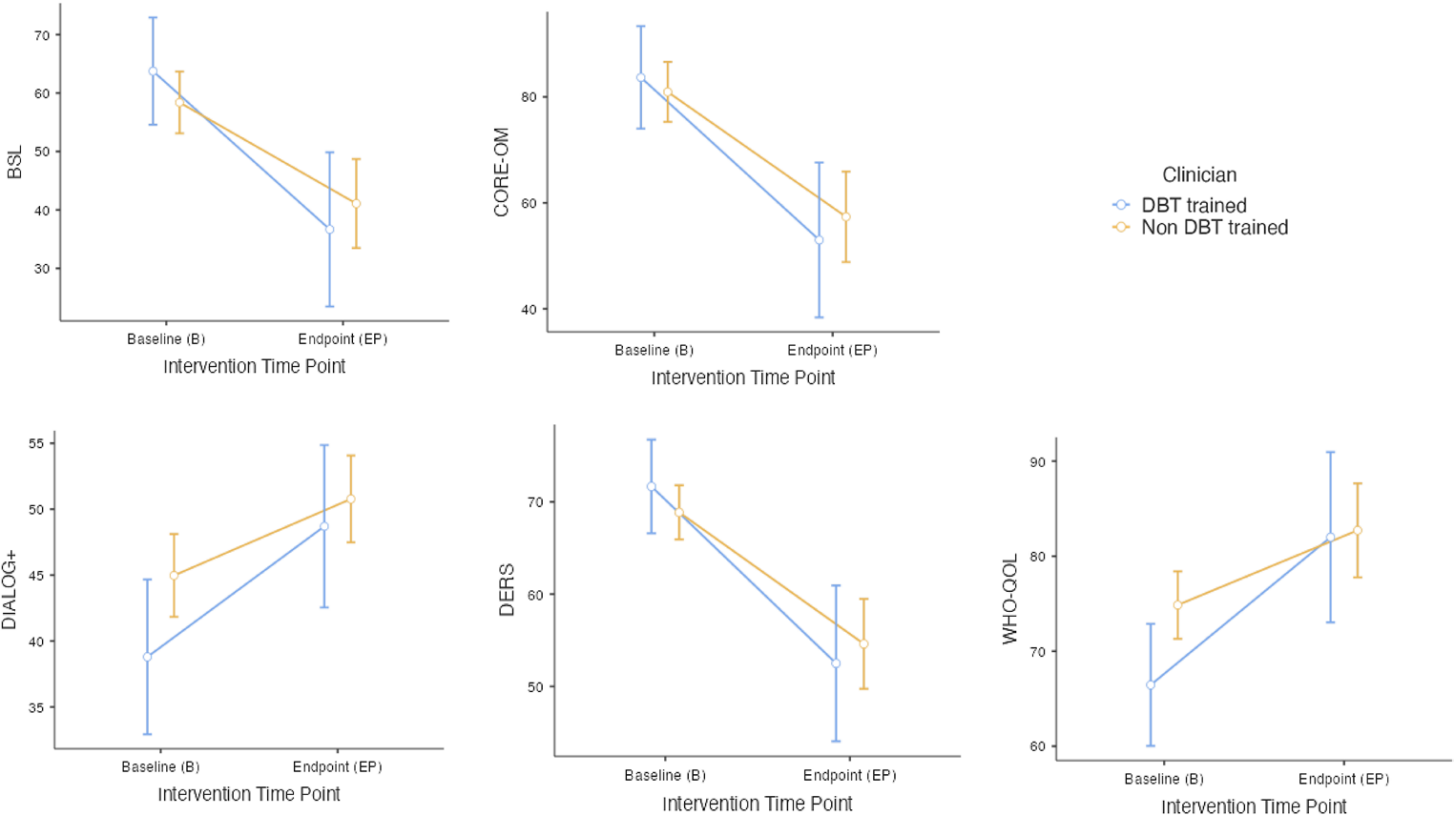
Mixed‐model ANOVAS showing the difference in mean baseline and endpoint scores on each outcome measure between fully DBT and non‐fully DBT‐trained clinicians.

To explore Aim 2, mixed‐model ANOVAs revealed relevant reductions and increases in mean scores from baseline to endpoint across all outcome measures, within both the fully DBT‐trained and non‐fully DBT‐trained clinician groups (see Figure 2). Despite this, these groups were not statistically significantly different from each other (*p* > .05).

### Service‐related outcomes

To explore Aim 3, descriptive statistics revealed that 33 of the total 48 participants (68.75%) were ready for discharge post‐intervention; either, already having been discharged, being on the discharge pathway or being referred to a different service. Of the 48, 15 (31.25%) remained open to the youth services post‐intervention, where most (18.75%) were provided a further needs‐led intervention (e.g., another brief psychological intervention or stepped up to full‐programme DBT). Whilst the number of participants per each service‐related outcome differed by clinician group (Aim 4), the Fisher's Exact Test revealed this difference to be non‐significant (*p* = .292). See Table 3 for all service‐related outcomes.

**TABLE 3:** Post‐intervention service‐related outcomes, by clinician training.

Service‐related outcome	Participants (*n*, %)	Clinician training	
		Fully DBT trained (*n*, %)	Non‐fully DBT trained (*n*, %)
Ready for discharge	33 (68.75%)	10 (83.3%)	23 (63.9%)
Discharge pathway	27 (56.25%)		
Discharged from services	5 (10.42%)		
Onward referral	1 (2.08%)		
Remaining open to service	15 (31.25%)	2 (16.7%)	13 (36.1%)
Medication management	1 (2.08%)		
Risk management	2 (4.17%)		
Second IDEAS episode	2 (4.17%)		
Other further intervention	9 (18.75%)		
Not specified	1 (2.08%)		

*Note*: Participant ‘*n*’ is out of the total number of participants who were included in the evaluation (*n* = 48).

## DISCUSSION

To the authors' knowledge, this small‐scale service evaluation is the first to examine a DBT‐ and MBT‐informed brief psychological intervention for a diagnostically heterogeneous group of young people with moderate CEN. Early exploratory analyses revealed symptom change across all time points (RQ1), as revealed within both clinician groups (RQ2). Further, a greater proportion of participants were ready for discharge post‐intervention (RQ3), irrespective of clinician training (RQ4).

The preliminary findings suggest participants experienced significant reductions in borderline symptom severity, psychological distress and emotion dysregulation, alongside improvements in quality of life and overall life satisfaction from baseline to endpoint. This highlights how IDEAS was effective in producing broad psychological and wellbeing benefits specific to this youth population. Although a smaller sample provided follow‐up data, the pattern of sustained change suggests that such gains were largely maintained at 3‐month follow‐up, albeit with smaller effect sizes. This attenuation may reflect the natural stabilization of treatment effects post intervention completion, as noted to occur post psychological therapy (Barkham & Lambert, 2021) and post DBT treatment (Linehan et al., 2006). Overall, such outcomes likely reflect IDEAS' grounding in third‐wave, DBT‐informed principles that target transdiagnostic processes (e.g., emotion dysregulation) through structured and individualized skills‐based learning, with the encouragement of real‐world application; strengths of the intervention that are crucial in preventing later symptom escalation and “revolving door” patterns (Menzies et al., 2024). More participants were therefore ready for discharge post‐intervention, supporting service capacity and waiting lists. For those not discharged, IDEAS identified outstanding needs to guide ongoing support and reduce time spent on waiting lists without targeted care, thereby lowering the risk of unmet needs escalating.

Interestingly, baseline to endpoint symptom change was not influenced by clinician training. Whilst a steeper drop was seen within the fully DBT‐trained clinician group, this was not significantly different from the non‐fully DBT‐trained group. This could suggest that costly, formalized training for clinicians (e.g., full DBT training) is not necessarily required to deliver IDEAS effectively and obtain positive outcomes, suggesting its cost‐efficacy. Indeed, such interpretations must be considered within the context of the small and unequal clinician group sizes, and the potentially non‐complete randomisation of participants to clinicians. The economic implications of IDEAS must therefore be more rigorously explored against clinician characteristics (e.g., years of experience in service and/or working with population); however, its potential cost value to services is noted.

Notably, service‐user dropout affected the delivery and completion of IDEAS. Here, 22 participants (26.5%) of the total 83 who received IDEAS dropped out before the minimum four session requirement. Anecdotal observations for this include inappropriate initial referrals leading to its delivery to unsuitable participants, resulting in its perceived irrelevance and subsequent dropout (Kazdin et al., 1997). Research further notes the complexity of service‐users' lives as a factor in secondary mental health care (Ruggeri et al., 2007), making dropout common in youth with severe and enduring mental health difficulties (De Soet et al., 2023; Warren et al., 2010). Indeed, research reports attrition rates of between 27% and 75% within general youth mental health care treatment (Baruch et al., 2009; De Haan et al., 2013; Luk et al., 2001). These do, however, vary per treatment received, where intervention for depression sees lower rates (14% for younger adolescents and 33% for older adolescents; Rohden et al., 2017; Wright et al., 2021) compared to those for DBT (17%–39%; Koons et al., 2001; Linehan et al., 1991; Linehan et al., 2006; Linehan et al., 2002; Linehan et al., 1999; McMain et al., 2009; Verheul et al., 2003), particularly DBT in the community (24%–58%; Feigenbaum et al., 2012; Priebe et al., 2012; Turner, 2000). The attrition rates of 26.5% observed within IDEAs therefore compares favourably to the literature, suggesting it demonstrating good engagement and acceptability relative to comparable youth treatments. This may speak to the selection criteria framework of IDEAS, which, through its broader scope, identified more suitable participants over those solely using narrower, fixed diagnostic classifications (Broersen et al., 2020). Alternatively, it could speak to the strengths of IDEAS in correctly targetting and meeting the needs of individuals (e.g., through its structured and individualized skills sessions) or the positive therapeutic relationship between clinician and service‐user; factors noted as key for treatment engagement within often reluctant youth (De Soet et al., 2023). Here, the supervision model that IDEAS adopts serves considerable purpose in supporting implementation and monitoring participants with high initial symptom severity, for whom dropout may be higher (Wright et al., 2020); a further strength of the intervention that may have contributed to the lower attrition rates.

### Strengths and limitations

This pilot was conducted as part of routine clinical practice with real‐world service‐users and is therefore an ecologically valid representation of a brief psychological intervention for CEN. Its co‐development with experts by experience, alongside its person‐centred and individualized skills sessions, results in the appropriate targetting of the needs of young people with CEN whilst also offering structure for clinicians to support treatment delivery to this population. Several limitations do however warrant caution of the findings.

First, IDEAS is in the pilot stage and was delivered as a service evaluation rather than a full research project. Consequently, it lacked a comparative control group (e.g., a waitlist control or a group from another service or Trust), limiting certainty that observed outcomes were directly attributable to the intervention. This is particularly relevant given the developmental context of the participants, whereby emotion dysregulation may decrease over time due to natural biological maturation, independent of treatment (Bunford & Evans, 2017). Should IDEAS progress to a formal clinical trial (including an appropriate control group), causal inferences regarding its efficacy would be strengthened. Second, participants were from youth services in East Anglia and were predominantly of a particular demographic (e.g., female, White). Generalisability of the findings to more diverse populations is thus limited. Third, all outcomes relied on self‐report, introducing potential response bias and reducing reliability and validity of the findings. This is particularly for measure items exploring coping strategies that can be interpreted variably within this age group (e.g., alcohol use). Fourth, the attrition observed poses the possibility that those providing endpoint and 3‐month data represent a subgroup with more favourable outcomes, introducing potential bias to the findings and reduced representation of the true population group. Fifth, unequal numbers of fully DBT‐trained and non‐fully DBT‐trained clinicians delivered IDEAS, limiting conclusions about training effects on outcomes; an area the future research must more rigorously explore. Finally, given the pilot nature, several outcomes were not assessed, such as adverse events and safety monitoring, participant satisfaction and acceptability, economic costs and benefits and follow‐up beyond 3 months; key priorities for future research.

### Clinical implications and future research

For young people, IDEAS can provide timely, responsive and therapeutic support that will help to prevent escalation of needs whilst sat on waiting lists, in crisis or prior to a potential diagnosis of PD. IDEAS seeks to recognize the heterogeneity of CEN presentations and to offer an individualized, flexible, non‐stigmatizing and collaborative approach; features consistent with ‘relational practice’, which has been associated with improved engagement in similar populations (Centre for Mental Health, 2022). For clinicians, IDEAS offers a structured framework for supporting young people with CEN, potentially reducing feelings of overwhelm and promoting skill development through training, supervision and the use of co‐produced guides and workbooks (Dickens et al., 2016). At service level, it can help maintain throughput of service‐users by facilitating discharge or onward referral, thus helping to reduce waiting times and overall clinical demand (Evans et al., 2017; NHS England, 2019c). On an organizational level, IDEAS may offer a lower‐cost complement to more intensive therapies (e.g., full DBT or MBT), given the potential lower level of training required by clinicians.

Yet, given its pilot stage, further exploration is warranted to formally explore its efficacy. Future research should include a control group to establish the effects of the intervention more rigorously. This could be done via comparison with generic group work, care coordination or other therapy groups that are not evidenced though conducted due to service constraints. Moreover, a longer follow‐up period could be included to explore the longer‐term maintenance of outcomes. Further, its economic value could be established in formal cost‐effectiveness analyses or simply by ensuring equal samples of clinicians of differing training. Finally, explorations of outcomes across services in wider regions, across diverse participant demographics and across clinician specialties, would be of utility to explore the efficacy of IDEAS on a wider scale.

## CONCLUSIONS

The current evaluation offers preliminary evidence of the benefits that IDEAS holds for young people, services and Trusts. As a brief psychological intervention, early exploratory analyses reveal its feasibility and potential lower cost, with the ability to enhance the treatment pathway for those presenting with, and supporting, those with moderate CEN. While not replacing formal psychological therapy for higher levels of need, its stepped‐care model provides a structured, accessible option within a wider continuum of support. Given the exploratory nature of these early findings, robust evidence using the aforementioned recommendations is required.

## AUTHOR CONTRIBUTIONS

**Annabel Harding:** Writing – original draft; conceptualization; methodology; formal analysis. **Franco Orsucci:** Conceptualization; funding acquisition; writing – review and editing; visualization. **Joanna Baines:** Conceptualization; writing – review and editing; methodology; supervision; resources; visualization.

## CONFLICT OF INTEREST STATEMENT

No authors have any interests to declare.

## TRANSPARENCY DECLARATION

The lead author affirms that the manuscript is an honest, accurate and transparent account of the study that is being reported, that no important aspects of the study have been omitted and that any discrepancies have been explained.

## Data Availability

The data that support the findings of this study are available from the corresponding author upon reasonable request.

## APPENDIX A FULL DEFINITIONS OF TERMS

1. ‘Youth Plus’: This is a needs‐type that can be used by mental health services to reflect young people navigating through ‘typical’ age‐related challenges, though they are demonstrating behaviour or responses that start to challenge or move beyond typical age‐expected norms. This could be related to a level of system upheaval (e.g., difficulties at home and/or at school), or a level of emotional dysregulation within the individual that requires a supportive approach to develop coping and regulation strategies and to manage teenage/youth life. Intervention for this group of young people is of a lower level (mild‐to‐moderate) than what may be required for those who fall under other needs‐types, though it is still required to be timely and responsive in order to avoid a shift into a more consistent presentation of emotional flux.
2. ‘Emotional Flux’: This is a needs‐type that can be used by mental health services to reflect a group of young people who present as emotionally overwhelmed and who are likely to move in and out of ‘crisis’ as a result of managing emotions through behaviour, such as through self‐harm. This is likely to be related to a level of system upheaval and/or individual emotional dysregulation, which contributes to a range of help‐seeking behaviours. Such young people who fall into this needs‐type are likely to have a history of early invalidating environments and a primary needs focus on safety, containment, validation and consistent engagement.
